# Preparation and Characterization of Hydroxyapatite Coating on AZ31 Mg Alloy for Implant Applications

**DOI:** 10.1155/2013/175756

**Published:** 2013-02-21

**Authors:** S. A. Salman, K. Kuroda, M. Okido

**Affiliations:** ^1^EcoTopia Science Institute, Nagoya University, Furo-cho, Chikusa, Nagoya 464-8603, Japan; ^2^Graduate School of Engineering, Al-Azhar University, Nasr City, Cairo 11371, Egypt

## Abstract

Magnesium alloys as biodegradable metal implants in orthopaedic research received a lot of interest in recent years. They have attractive biological properties including being essential to human metabolism, biocompatibility, and biodegradability. However, magnesium can corrode too rapidly in the high-chloride environment of the physiological system, loosing mechanical integrity before the tissue has sufficiently healed. Hydroxyapatite (HAp) coating was proposed to decrease the corrosion rate and improve the bioactivity of magnesium alloy. Apatite has been cathodically deposited on the surface of Mg alloy from solution that composed of 3 mM Ca(H_2_PO_4_)_2_ and 7 mM CaCl_2_ at various applied potentials. The growing of HAp was confirmed on the surface of the coatings after immersion in SBF solution for 7 days. The coating obtained at −1.4 V showed higher corrosion resistance with bioactive behaviors.

## 1. Introduction

Metal materials, including stainless steels, titanium, and cobalt-chromium-based alloys, are commonly used for implant devices due to their high strength, ductility, and good anticorrosion properties [1]. It is more suitable for load-bearing applications compared with ceramics or polymeric materials due to their combination of high mechanical strength and fracture toughness [2]. The release of toxic metallic ions or particles by corrosion or wear processes leads to undesirable effects on cell and bone tissues [3]. Moreover, these metallic materials are not biodegradable in the human body and can cause long-term complication (infection) [4]. The elastic modules of current metallic biomaterials are not well matched with that of natural bone tissue, resulting in stress shielding effects that can lead to reduced stimulation of new bone growth and remodeling which decreases implant stability [5]. Comparing to commonly approved metallic biomaterials, magnesium alloys have many outstanding advantages due to their attractive biological property including being essential to human metabolism, biocompatibility, and biodegradability [6].

The mechanical properties of magnesium alloys are similar to those of natural bone (40–57 GPa) [7]. Moreover, magnesium is one of the most important bivalent ions associated with the formation of biological appetites and plays an important role in the changes in the bone matrix that determines bone fragility [8]. On the other hand, implants made of magnesium alloys were degraded in vivo, eliminating the need for a second operation for implant removal. Good biocompatibility was observed in clinical studies [9]. Unfortunately, magnesium can corrode too rapidly in the physiological pH (7.4–7.6) and high-chloride environment of the physiological system, loosing mechanical integrity before the tissue has sufficiently healed and producing hydrogen gas in the corrosion process at a rate that is too fast to be dealt with by the host tissue [10].

Recently, some researches have been done to slow down the biodegradation rate of magnesium alloys, including fluoride conversion coating [11], alkali-heat treatment [12], and plasma immersion ion implantation [13]. Besides improving the biodegradation rate of magnesium alloys, the biocompatibility should also be considered. The hydroxyapatite [Ca_10_(PO_4_)_6_(OH)_2_], hereafter (HAp), coating can satisfy the dual properties. Synthetic HAp ceramics was routinely used as porous implants, powders, and coatings on metallic prostheses to provide bioactive fixation. The presence of sparingly soluble HAp coatings led to bone tissue response (osteoconduction) in which bone grew along the coating and formed a mechanically strong interface [14]. Therefore, HAp coating was deposited on the surface of metallic implants to improve the biocompatibility property and to decrease the degradation rate in the physiological environment. Deposition of HAp coatings has been achieved by a number of methods, including plasma spraying [15], ion implantation [16], sputtering [17], sol-gel coating [18], biomimetic methods [19], electrophoretic deposition (EPD) [20], electrochemical deposition [21], and electrospray deposition (ESD) [22, 23].

Among these methods, electrochemical deposition is known to be simple, flexible, and inexpensive technique for fabricating the metallic thin films and does not require complex and expensive vacuum apparatus. In addition, the deposition processing can be conducted at room temperature, and the morphology of coating can be controlled easily by varying the electrochemical potential and electrolyte concentration [24].

In this study, electrodeposition method was employed to produce HAp coating on AZ31 magnesium alloy in order to improve the anticorrosion properties and to enhance its bioactivity. The electrodeposition process was performed in calcium phosphate electrolyte with other additives at optimized pH and temperature. The effect of applied potential on the structure and the anticorrosion properties was investigated. The anticorrosion properties of the developed coatings were evaluated by the anodic polarization and electrochemical impedance spectroscopy techniques. The surface morphology and phase structure of coated films were also examined using scanning electron microscope (SEM), X-ray diffraction (XRD), and Energy Dispersive Spectroscopy (EDS). The HAp coating could enhance the anticorrosion property and effectively improved in vitro bioactivity in simulated body fluid.

## 2. Materials and Methods

### 2.1. Specimens Preparation

Commercially available AZ31 Mg alloy (3 mass% Al, 1 mass% Zn) was used as the substrate. The chemical composition of the alloy is listed in Table 1. The surface of the alloys was polished up to no. 2000 emery paper followed by 0.05 *μ*m alumina powders. The specimens were carefully cleaned with water, rinsed with acetone, and dried under air. All of the experiment specimens were mounted using polytetrafluoroethylene (PTFE) resin tape leaving and exposed surface area of 1 cm^2^.

### 2.2. The Film Formation

The specimens were immersed in 3 mM Ca(H_2_PO_4_)_2_ + 7 mM CaCl_2_ solution at 37°C and pH 6 for 30 min. The electrodeposition was performed at −1.4, −1.6, and −1.8 V applied potentials using a conventional electrochemical cell equipped with three electrodes. Platinum, Ag/AgCl sat. KCl, and magnesium alloy specimen served as the counter, the reference, and the working electrode, respectively.

### 2.3. Characterization Methods

The morphology and microstructure of the films were observed with Hitachi S-800 scanning electron microscope (SEM). The crystal structure of the coated films was identified using thin film X-ray diffraction (XRD) analysis using a Shimadzu XRD-6000 X-ray diffractometer with Cu K*α* radiation with scan step 0.02° and scan speed 2°/min. from diffraction angle of 10 to 60 at 30 kV and 20 mA. The elemental composition of the coating was identified using Energy Dispersive Spectroscopy (EDS).

### 2.4. Anticorrosion Measurements

The anodic polarization and electrochemical impedance spectroscopy tests were carried out using a Solartron 1285 Potentiostat from Solartron Analytical, Farnborough, United Kingdom. The measurements were controlled by Scribner Associates Corrware and Z polt electrochemical experiment software, respectively. The polarization curves were measured in phosphate buffered saline PBS(−) solutions with a scanning rate of 1 mV/s. 

### 2.5. Bioactivity Experiment in Simulated Body Fluid

The coating was immersed for 7 days in the SBF (Kokubo solution) with ion concentrations nearly the same as those of the body blood plasma. The pH of the SBF solution was adjusted to 7.4. The phase structure, surface morphology, and the anticorrosion property were examined every day by removing the specimens from SBF solution, rinsed with distilled water, and air-dried. 

## 3. Results and Discussion

High bioactivity can be achieved by performing the coating in the condition that is near to the human internal environment. Some research groups have applied various ranges of temperatures (room temperature; 45, 50, and 80°C) and initial pH (5.8, 6.5, and neutral) [25].

To achieve pure apatite coating on magnesium alloy, the initial pH and temperature should be carefully chosen. In our previous work, the optimum solution pH and temperature were defined [26]. The HAp peaks could be observed only at the temperature of 37°C or higher. It has been reported that the growth rate of apatite layer increased with the increasing temperature due to the decreasing in the solubility product of HAp [27]. 

HAp coating was deposited on the AZ31 alloy using the electrodeposition method. Fixed potential was set to −1.4, −1.6, and −1.8 V, so that the Mg alloy substrate became negatively charged (as a cathode). Magnesium dissolution takes place immediately after immersion of the specimen in 3 mM Ca(H_2_PO_4_)_2_ and 7 mM CaCl_2_ solution for 30 min at pH 6. according to reaction (1). Water is reduced at the cathode surface to produce hydrogen gas and hydroxide ions, which will lead to an increase in the solution pH according to reaction (2):

(1) $$\begin{array}{c} \text{Mg}={\text{Mg}}^{2+}+2{\text{e}}^{-} \end{array}$$

(2) $$\begin{array}{c} 2{\text{H}}_{2}\text{O}+2{\text{e}}^{-}=2{\text{OH}}^{-}+\text{H} \end{array}$$

**Figure 1:**
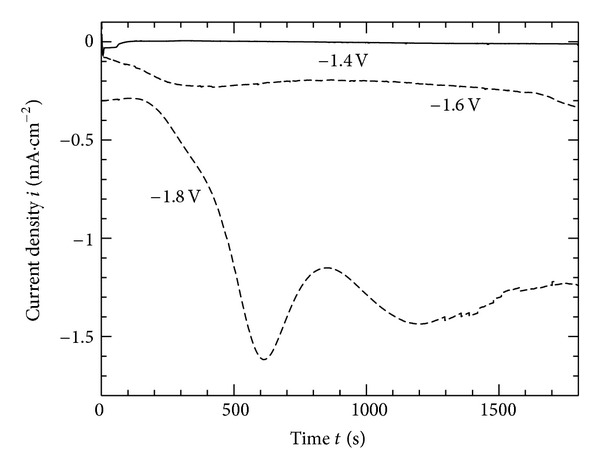
Current change during cathodic electrodeposition on AZ31 Mg alloy in 3 mM Ca(H_2_PO_4_)_2_, 7 mM CaCl_2_ for 30 min.

Figure 1 shows the current-time curves during the electrodeposition treatment at various potentials. At −1.8 V, the current density sharply decreased indicating the early formation of the coating film. On the other hand, the current density has a slight decrease at −1.6 V and has nearly a stable rate at −1.4. The local increase in the pH value and availability of Ca and P ions in the solution are expected to encourage the deposition of HAp and dicalcium phosphate dihydrate [CaHPO_4_ · 2H_2_O], hereafter (DCPD) on the magnesium alloy surface as shown in reactions (3) and (4):

(3) $$\begin{array}{c} 5{\text{Ca}}^{2+}+3{\text{PO}}_{4}+{\text{OH}}^{-}\to \frac{1}{2}\left({\text{Ca}}_{10}{\left({\text{PO}}_{4}\right)}_{6}{\left(\text{OH}\right)}_{2}\right) \end{array}$$

(4) $$\begin{array}{c} {\text{Ca}}^{2+}+\text{H}{\text{PO}}_{4}+2{\text{H}}_{2}\text{O}\to {\text{CaHPO}}_{4}\cdot 2{\text{H}}_{2}\text{O} \end{array}$$

**Figure 2:**
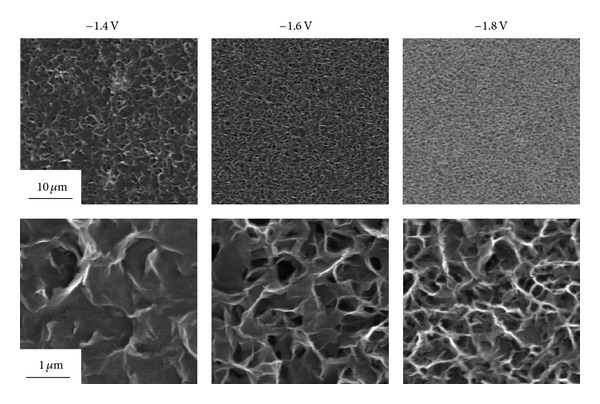
SEM images of coating films formed on AZ31 Mg alloy in 3 mM Ca(H_2_PO_4_)_2_, 7 mM CaCl_2_ for 30 min. at −1.4 V, −1.6 V, and −1.8 V.

Figure 2 shows the SEM images of coating films formed at −1.4 V, −1.6 V, and −1.8 V. Flake-like particles were observed on all surfaces after cathodic deposition, while the size of crystallites becomes finer with increasing the cathodic potential to more negative side. 

**Figure 3:**
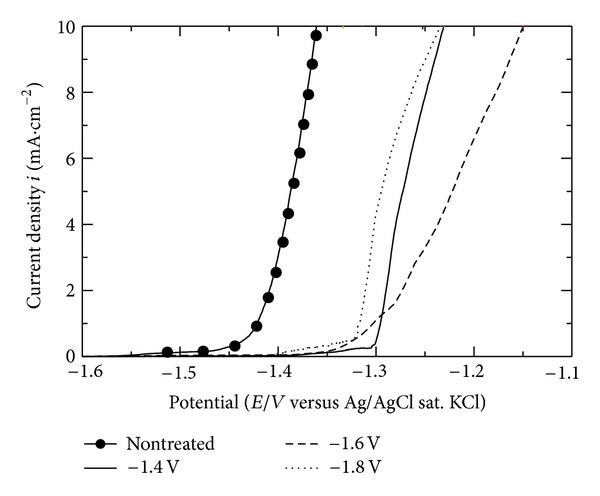
Anodic polarization test in PBS(−) for AZ31 magnesium alloys before and after treatment at −1.4 V, −1.6 V, and −1.8 V.

The corrosion resistance of the coating films formed at −1.4, −1.6, and −1.8 V was examined in PBS(−) solution using anodic polarization tests as shown in Figure 3. The results show that the corrosion reaction does not take place easily as it indicated by the decrease of corrosion currents and the increase of polarization resistances. The pitting potential of all Hap-coated specimens shifted toward the noble direction in comparison to the nontreated specimen. This indicates that the coating films offered a corrosion protection, and Mg alloy was able to be prevented from being dissolved. Moreover, the coating obtained at −1.4 V has the nobler pitting potential. 

**Figure 4:**
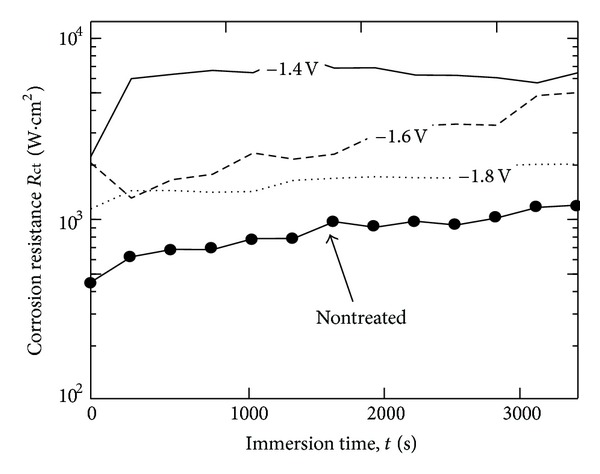
Change in Rct in PBS(−) for AZ31 magnesium alloys before and after treatment at −1.4 V, −1.6 V, and −1.8 V.

EIS test was performed to ensure the best corrosion resistance film. Corrosion rate determination is associated with the charge transfer resistance (Rct) by using EIS technique. The charge transfer resistance is equal to the diameter of the semicircle in the complex plane graph (Nyquist diagram).  Figure 4 shows the time dependence of the charge transfer resistance, which corresponds to corrosion rate. The charge transfer resistance increases by the treatment in 3 mM Ca(H_2_PO_4_)_2_ and 7 mM CaCl_2_ solution at various applied potentials. The film formed at −1.4 V has much higher Rct value than those formed at −1.8 and −1.6 V. This result is in agreement with anodic polarization test which indicates that the film formed at −1.4 V has the best anticorrosion properties.

Improving corrosion resistance and decreasing dissolution rate of implant materials are highly recommended to allow the bone to heal. Therefore, we performed the bioactivity experiments on the coating obtained at −1.4 V. The in vitro examination was carried out at 37°C for 7 days in order to evaluate the bioactive behaviors of HAp coating in SBF solution, which has the ion element equal to or close to that of blood plasma. 

**Figure 5:**
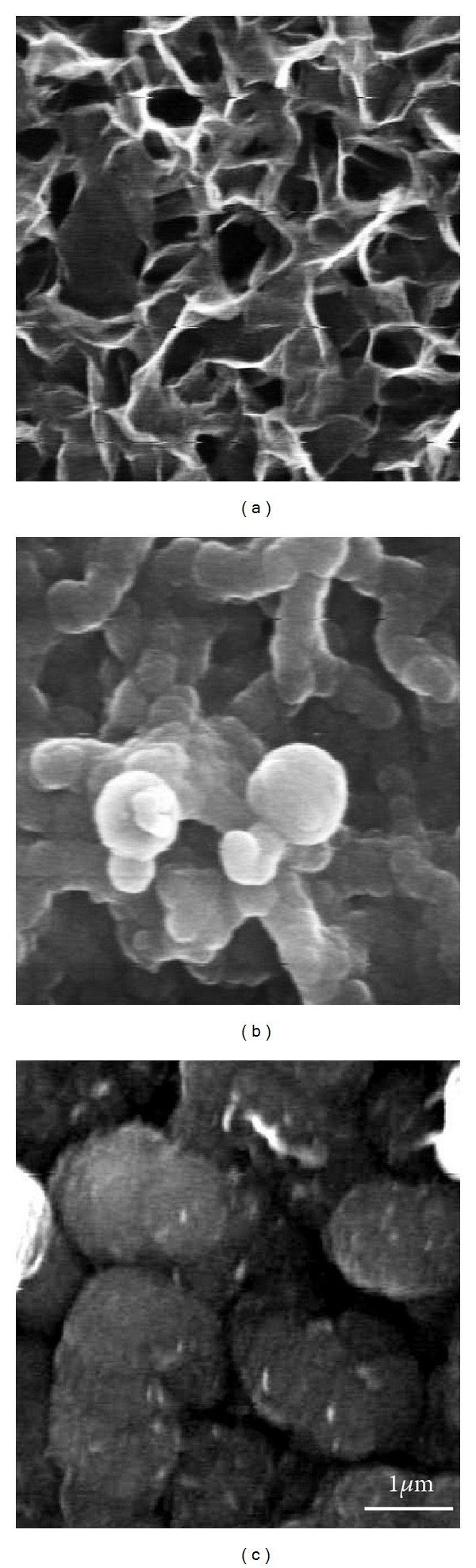
SEM images of coating obtained at −1.4 V before and after immersion in SBF solution for 1 day and 7 days.

Figure 5 shows the surface morphologies of the coating obtained at −1.4 before and after immersion in SBF solution for 1 day and 7 days. The growing of HAp was confirmed on the surface of the coating after immersion for 1 day in SBF solution. The particles sizes were finer upon immersion in SBF solution. The growing rate of HAp on the surface of the coating is notably increased with increasing the immersion time to 7 days.

**Figure 6:**
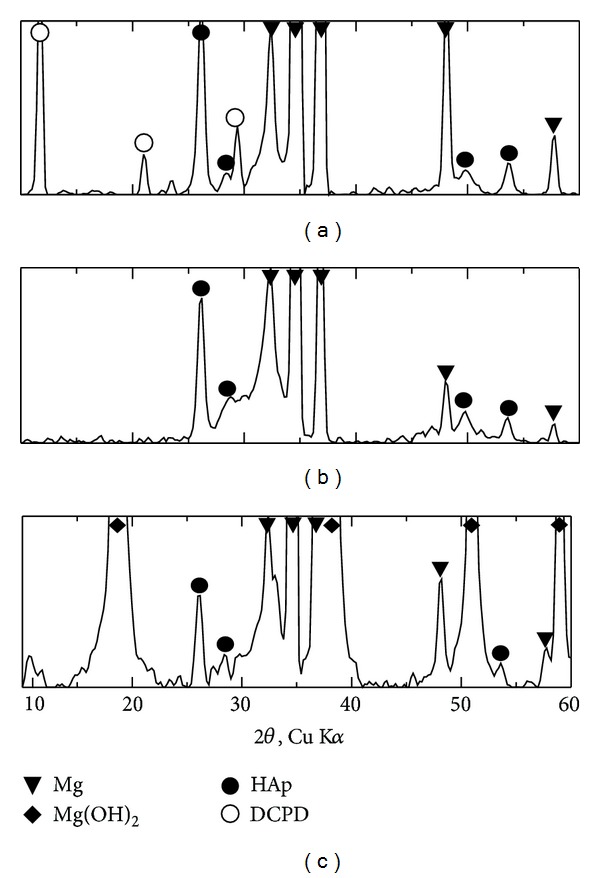
xrd patterns of coatings obtained at −1.4 V before and after immersion in SBF solution for 1 day and 7 days.

Figure 6 shows the XRD results of the coating obtained at −1.4 before and after immersion in SBF solution for 1 day and 7 days. The DCPD and HAp peaks were seen before immersion in SBF solution.

With immersion in SBF solution, DCPD was converted into more stable HAp. With increasing of immersion time to 7 days, HAp peaks have increased which confirms the growth of HAp 

**Figure 7:**
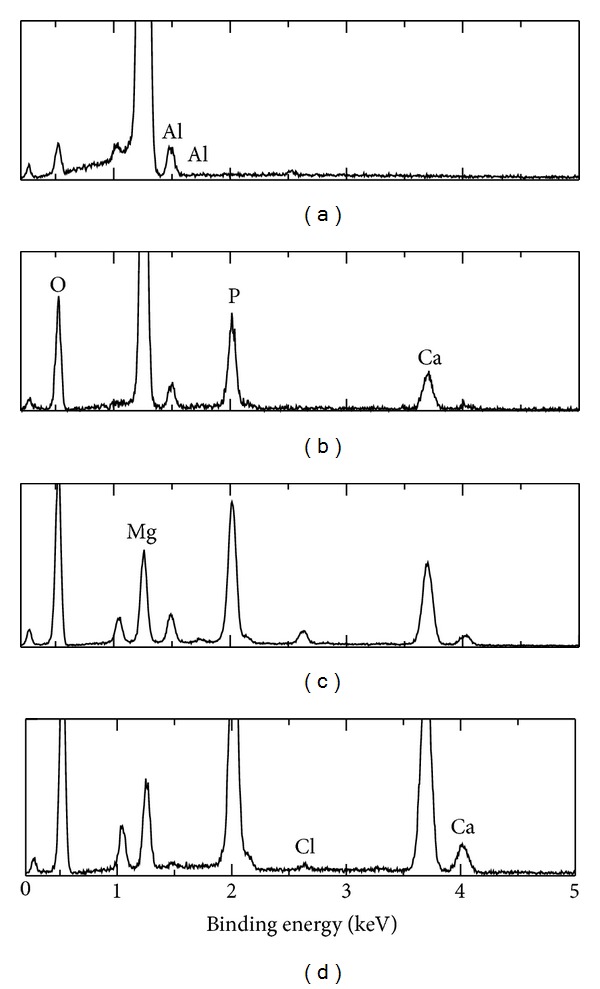
EDS patterns of coatings obtained at −1.4 V before and after immersion in SBF solution for 1 day and 7 days.

Figure 7 shows the EDS results of the coating obtained at −1.4 before and after immersion in SBF solution for 1 day and 7 days. Strong peaks of calcium (Ca), phosphorous (P), oxygen (O), magnesium (Mg), aluminum (Al), and several small peaks were observed before and after immersion in SBF solution.  Figure 6(a) shows that the coating film composed of Mg, Al, and calcium phosphate compounds which are identified as HAp and DCPD from previous XRD test. With immersion in SBF solution for 1 day, the peaks of Ca and P were increased and Mg peaks were decreased which indicate growing of HAp as shown in Figure 6(b). With increasing of immersion time to 7 days, the peaks of Ca and P continued to increase and Mg peaks continued to decrease as shown in Figure 6(b). This continuous growing of HAp with immersion for 7 days, obviously confirms the bioactivity of this coating 

## 4. Conclusions

The hydroxyapatite coating was successfully electrodeposited on magnesium alloy surface by immersing in 3 mM Ca(H_2_PO_4_)_2_ and 7 mM CaCl_2_ solution for 30 min and at temperature of 37°C. The anticorrosion property of AZ31 magnesium alloy was improved with hydroxyapatite coating, and the best anticorrosion properties were obtained at −1.4 V. Hydroxyapatite was grown on the coating with immersion in SBF solution for 7 days, which proves its bioactivity.

## Figures and Tables

**Table 1:** Chemical composition (mass%) of AZ31 Mg alloy.

Al	Zn	Mn	Si	Cu	Ni	Fe	Mg
3.0	1.0	0.43	0.01	<0.01	<0.001	0.003	Bal.
